# Should I stay or should I go? Transsulfuration influences invasion and growth in glioblastoma

**DOI:** 10.1172/JCI176879

**Published:** 2024-02-01

**Authors:** András K. Ponti, Daniel J. Silver, Christopher Hine, Justin D. Lathia

**Affiliations:** 1 Department of Molecular Medicine, Cleveland Clinic Lerner College of Medicine and Case Western Reserve University, Cleveland, Ohio, USA.; 2 Department of Cardiovascular and Metabolic Sciences, Cleveland Clinic Lerner Research Institute, Cleveland, Ohio, USA.; 3 Case Comprehensive Cancer Center, Cleveland, Ohio, USA.; 4 Department of Pathology, Cleveland Clinic Lerner College of Medicine and Case Western Reserve University, Cleveland, Ohio, USA.

## Abstract

A major challenge in treating patients with glioblastoma is the inability to eliminate highly invasive cells with chemotherapy, radiation, or surgical resection. As cancer cells face the issue of replicating or invading neighboring tissue, they rewire their metabolism in a concerted effort to support necessary cellular processes and account for altered nutrient abundance. In this issue of the *JCI*, Garcia et al. compared an innovative 3D hydrogel–based invasion device to regional patient biopsies through a comprehensive multiomics-based approach paired with a CRISPR knockout screen. Their findings elucidate a role for cystathionine γ-lyase (CTH), an enzyme in the transsulfuration pathway, as a means of regulating the cellular response to oxidative stress. CTH-mediated conversion of cystathionine to cysteine was necessary for regulating reactive oxygen species to support invasion. Meanwhile, inhibition of CTH suppressed the invasive glioblastoma phenotype. However, inhibiting CTH resulted in a larger overall tumor mass. These findings suggest that targeting the transsulfuration pathway may serve as a means of redirecting glioblastoma to proliferate or invade.

## Glioblastoma treatment hampered by infiltrative nature

Glioblastoma (GBM) is the most common primary malignant brain tumor. Long-term survival rates remain well below those of many other cancer types. The reason behind such poor long-term survival is the inability to effectively resect or kill all tumor cells throughout the brain, resulting in rapid and inevitable disease reoccurrence. The many contributing factors include extensive cellular heterogeneity, an immune-suppressive tumor microenvironment, the blood-brain barrier, and the highly infiltrative nature of GBM (1, 2). Despite advances in diagnosis, surgical techniques, and radiochemotherapy, recurrence is almost always inevitable within the area surrounding the original tumor. This persistence is due to high brain penetrance of GBM cells through perivascular space around blood vessels and parenchymal space containing neurons as well as glial cells into the surrounding healthy tissue (3). Targeting invading cells proves extremely difficult regardless of treatment modality once they have escaped beyond the reach of the radiation field and what is considered maximally safe in terms of surgical margins. Therefore, it is imperative to identify molecular mechanisms contributing to and supporting the highly invasive nature of GBM.

Research in the field has identified the presence of GBM stem cells (GSCs) along with altered cellular functions including neural circuit and extracellular matrix remodeling, rearrangement of cytoskeletal proteins, and activation of pathways involved in invasion. GSCs located along the infiltrative edge possess a high degree of phenotypic plasticity that enables them to maintain fitness as they invade through different zones of growth within the brain (4, 5). Meanwhile, tumor cells functionally hijack and rewire neuronal signaling to establish a communicative multicellular network through the use of tumor microtubes (6–8). Additionally, GBM invasion is driven by the formation of migratory invadopodia and filopodia, along with upregulation of matrix remodeling proteins, and activation of pathways found to induce an epithelial-mesenchymal transition (9–11). While many such pathways are responsible for driving the invasive phenotype of GBM, metabolic requirements must be met to support cellular energetic demands.

## CTH balances levels of ROS required for invasion

**Figure 1:**
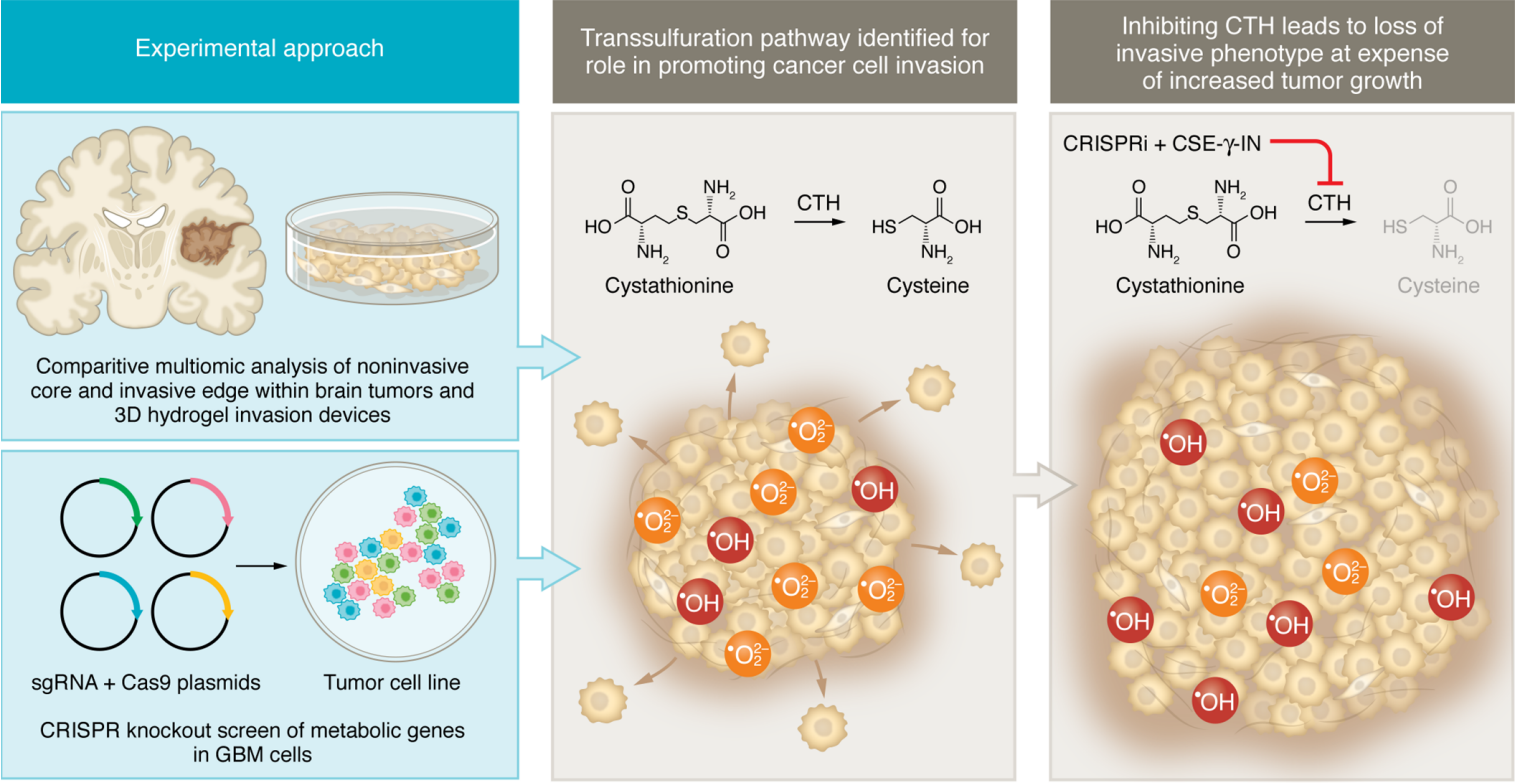
Multiomics analysis indicates the importance of CTH in GBM brain infiltration. Metabolomics, lipidomics, RNA-Seq, and a CRISPR knockout screen identify CTH-mediated cysteine production as a potent regulator of oxidative stress–induced invasive potential in GBM. Samples along the invasive tumor front show elevated levels of CTH, cystathionine, and peroxide ROS. The absence of CTH leads to a reduction in cysteine production, resulting in an increased accumulation of hydroxyl radical ROS, which yields reduced brain infiltration while driving enhanced tumor growth. CSE-γ-IN, cystathionine-γ-lyase-IN-1.

Cells within the tumor switch from requiring abundant supplies of nutrients in the form of glucose for the purpose of rapidly proliferating to utilization of lipids, amino acids, and nucleotides as they migrate through surrounding microenvironments with unique demands (12–14). To evaluate metabolic changes occurring in GBM invasion, Garcia et al., as reported in this issue of the *JCI*, examined cells isolated from the tumor core as well as from the invasive front of their 3D hydrogel invasion device and compared them with matching site-directed patient biopsies. Identification of genes responsible for the observed changes in metabolism was performed with the use of a CRISPR screen (Figure 1). Validation of their findings was performed using in vivo models to assess effects on tumor growth and invasion (15). This comparative analysis using metabolomics and lipidomics approaches identified the metabolite cystathionine as increased in both sample types. Both sets of invasive cells also expressed additional oxidative stress markers. Profiles of the changes responsible for the metabolic shift in invading GBM cells via RNA-Seq demonstrated an increase in genes involved in producing and responding to oxidative stress. These findings matched with an increase in abundance of reactive oxygen species (ROS) along the infiltrative edge.

Using a CRISPR knockout library of metabolic genes, Garcia et al. identified five genes linked to invasion. Among the five genes, pharmacological inhibition of only cystathionine γ-lyase (CTH, also known as CGL or CSE) was sufficient to inhibit the cellular invasive phenotype independently of proliferation. Upon CTH knockdown, the investigators did identify a population of cells that retained their invasive capabilities. Among these cells, it was observed that cystathionine β-synthase (CBS) was upregulated. This finding, paired with other recent studies identifying mercaptopyruvate transferase (MPST) activity being increased in GBM (16, 17), suggests that sulfur metabolism is tightly regulated. CTH, CBS, and MPST all function in the transsulfuration (TSS) pathway as enzymes responsible for processing homocysteine into its downstream metabolites, including cysteine, glutathione, pyruvate, and hydrogen sulfide (H_2_S). The combination of identifying increased cystathionine in invading cells and the inhibition of invasion that resulted from drugging CTH motivated the investigators to pursue further investigation of the TSS pathway for its handling of oxidative stress. While short-term exposure to peroxides promoted invasion, knockdown of CTH led to an accumulation of hydroxy radicals that were deleterious to the invading phenotype. When determining the mechanism by which CTH was involved in invasion, it was found that glutathione and H_2_S, both products of the TSS pathway, were not able to rescue the invasive phenotype lost under CTH knockdown. However, invasion could be rescued with exogenous cysteine. In vivo validation with CTH knockdown demonstrated decreased invasion; however, this change did not alter survival. Interestingly, in a previous edition of the *JCI*, Silver et al. demonstrated that CTH inhibition resulted in substantial GSC enrichment, increased tumor cell proliferation, and protection from necrotic cell death (18), all of which help to explain why overall survival was not improved with CTH knockdown. These results suggest a role for the TSS pathway and its metabolites as being important for regulating GBM migration versus growth.

## Implications and conclusions

Garcia et al. demonstrate a role for ROS mediation in GBM invasion (15). While oxidative stress and the handling of ROS have been reported as mediators of tissue invasion in other cancer types (19), Garcia and colleagues may be the first to extend this concept to GBM. It will therefore be intriguing to further investigate how oxidative stress can be managed as a means of preventing the highly infiltrative nature of GBM in patient care. Perhaps of greatest interest is understanding how this study adds to the growing body of literature that implicates the TSS pathway and its functional enzymes (i.e., CTH and CBS) as well as the metabolites utilized and produced by this pathway in GBM and in the broader field of cancer biology (20).

The TSS pathway provides an essential source of cysteine, glutathione, and the gaseous signaling molecule H_2_S. Equally important is the role of the TSS pathway in consuming upstream precursors to tightly regulate intracellular and extracellular levels of metabolites, such as homocysteine and cystathionine. Aberrant function of enzymes such as CTH and CBS has been linked to both pro- and antitumorigenic properties (21, 22). Similar to the dueling roles of the TSS pathway enzymes, metabolites in this pathway, such as H_2_S, have been found to either promote or suppress tumor growth (18, 23–25). Therefore, it seems increasingly likely that CTH functions and metabolic demands in GBM are very much context dependent and provide cues for when to “go” and when to “grow.”

When evaluating the role of CTH and its metabolites in GBM, spatial and functional assessment of gene expression within the tumor as well as stage of tumor growth should be considered. Notably, CTH-mediated production of H_2_S reduces tumor growth and GSC abundance (18), while cysteine production enables peroxide-dependent brain invasion (15). This work conducted by Garcia et al. (15) combined with what is currently known in the field may enable targeting of the TSS as a means of developing a multipronged approach for treating GBM.

## Version 1 02/01/2024

Electronic publication
